# Supplementary material for: Multilayered PVDF-HFP Porous Separator via Phase Separation and Selective Solvent Etching for High Voltage Lithium-Ion Batteries
Source: Membranes (Basel). 2021 Jan 7;11(1):41. doi: 10.3390/membranes11010041 (PMC7825726; doi:10.3390/membranes11010041)
Supplement: Supplementary file 1 [file membranes-11-00041-s001.pdf]

Supporting Information

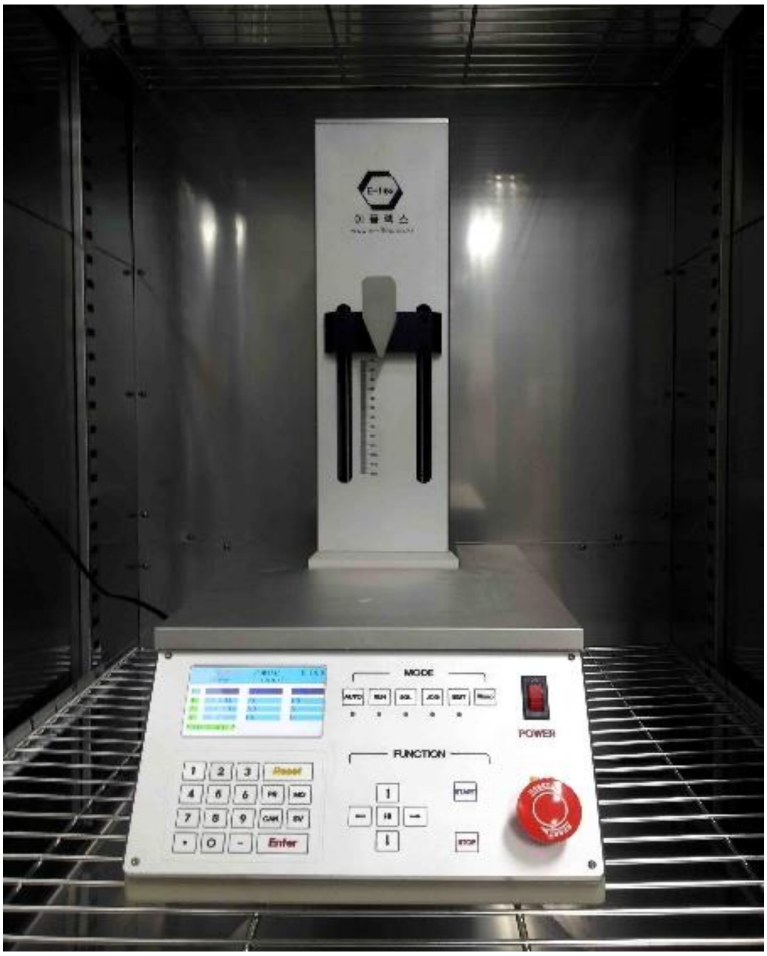

Figure S1. Photograph of the E-flex dip coater used in this study

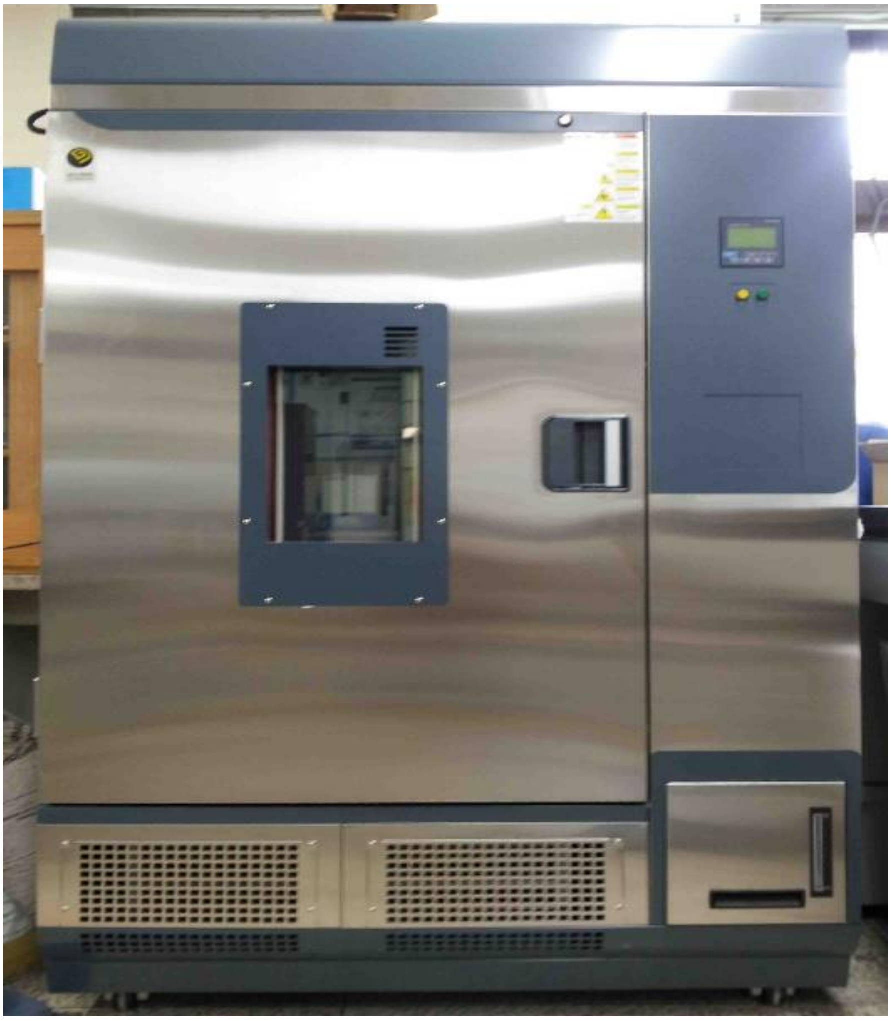

Figure S2. Photograph of the temperature and humidity controller.

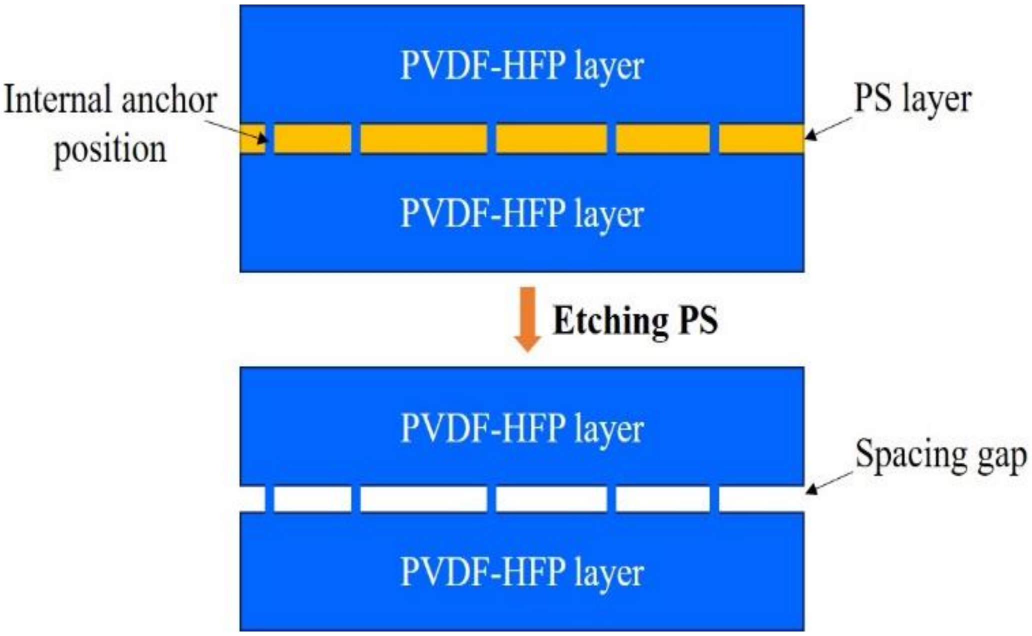

Scheme S1. Effect of the PS layer thickness on the adhesion between PVDF-HFP layers.

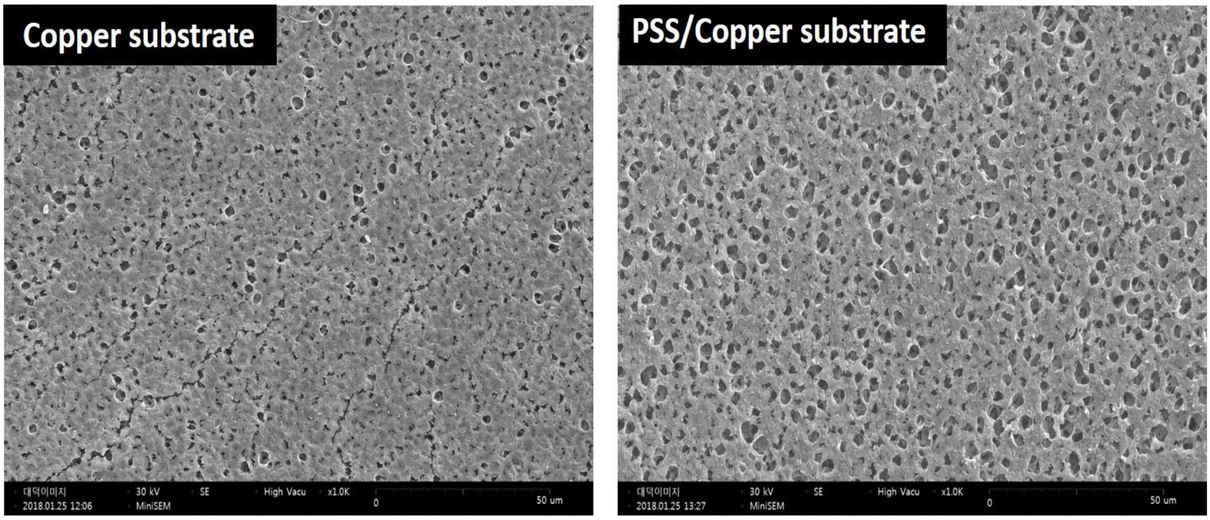

Figure S3. Surface morphology of the backside of the film (facing the substrate) using bare copper and PSS-coated copper substrates.

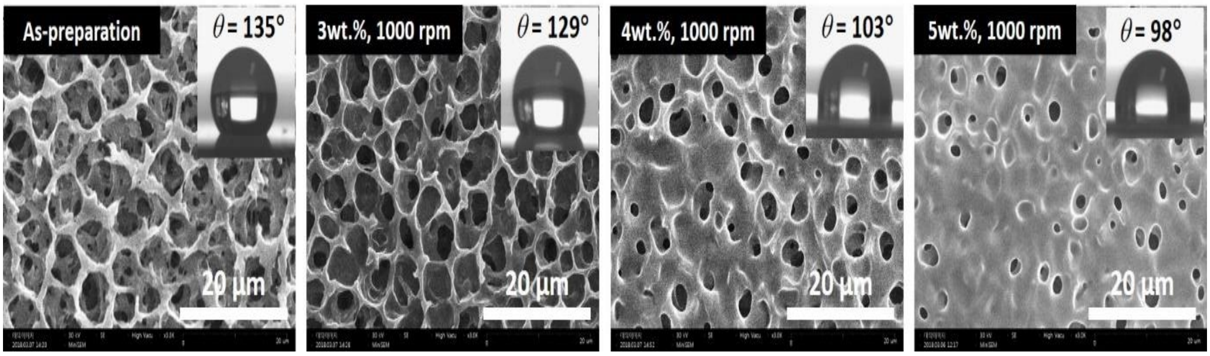

Figure S4. Surface morphology of the porous PVDF-HFP film after coating with a PS solution with different concentrations. The spin-coating was done at a rotational speed of 1000 rpm for 40 s.

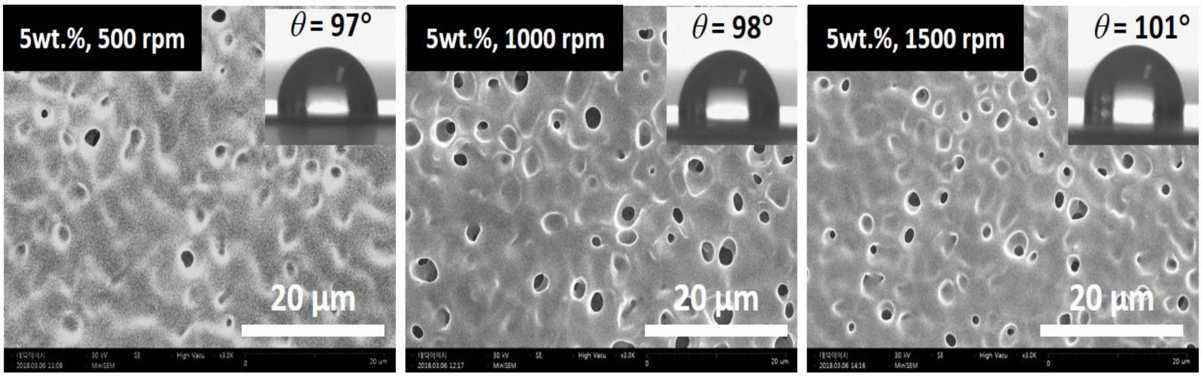

Figure S5. Surface morphology of the porous PVDF-HFP film after coating with a 5 wt.% PS solution at different rotational speeds.

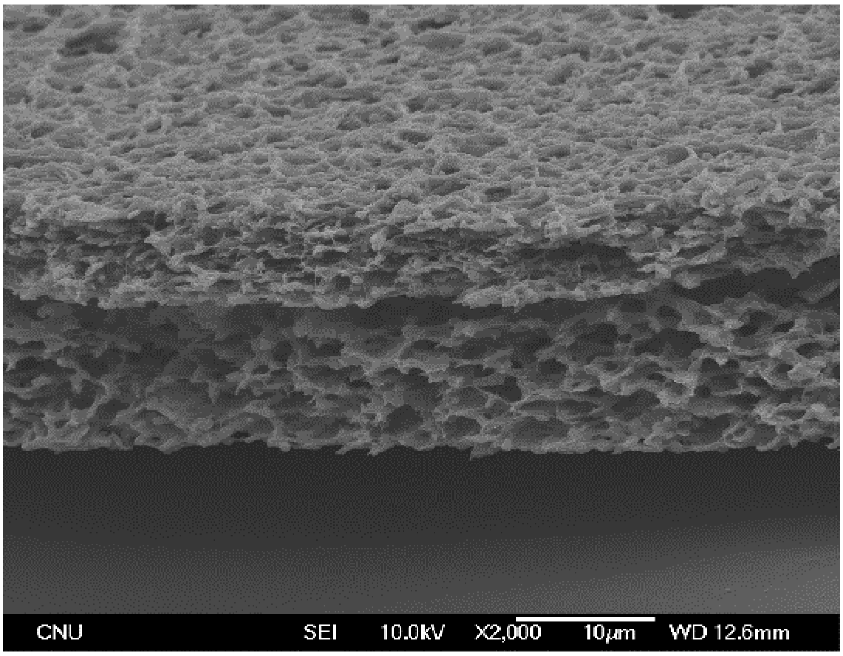

Figure S6. Cross-sectional morphology of the two-layer structured porous PVDF-HFP membrane prepared with a thicker PS coating layer.
